# Risk factors for antimicrobial-resistant *Enterobacterales* in dogs: a systematic review

**DOI:** 10.3389/fvets.2024.1447707

**Published:** 2024-10-07

**Authors:** Esa Karalliu, Kai Yeung Chung, Brett MacKinnon, Belete Haile, Pawel M. Beczkowski, Vanessa R. Barrs, Ibrahim Elsohaby, Omid Nekouei

**Affiliations:** ^1^Department of Infectious Diseases and Public Health, Jockey Club College of Veterinary Medicine and Life Sciences, City University of Hong Kong, Hong Kong, Hong Kong SAR, China; ^2^Department of Veterinary Clinical Sciences, Centre for Animal Health and Welfare, Jockey Club College of Veterinary Medicine and Life Sciences, City University of Hong Kong, Hong Kong, Hong Kong SAR, China

**Keywords:** antimicrobial resistance, risk factor, bacterial infection, dog, *Enterobacterales*

## Abstract

Identifying risk factors associated with the carriage of antimicrobial-resistant (AMR) bacteria in dogs is crucial to understanding their epidemiology and for developing and refining targeted control measures. However, relevant data is scattered and conflicting findings have been reported. This systematic review aimed to compile risk factors associated with the carriage of AMR-*Enterobacterales* in dogs worldwide and to identify relevant knowledge gaps for directing future research. A systematic review was conducted according to PRISMA guidelines, searching PubMed, CABi, and Scopus databases for studies reporting risk factors for acquiring AMR-*Enterobacterales* in dogs. After screening peer-reviewed, English-language studies by title/abstract, eligible studies were subjected to a full-text assessment, data extraction, risk-of-bias assessment, and qualitative synthesis. In the initial search, 774 articles were identified, including 274 duplicates. After screening by title/abstract, 77 articles were subjected to full-text review, from which 40 were eventually selected for data extraction, including 29 cross-sectional, six cohort, and five case-control studies. The most frequently investigated risk factors for AMR-*Enterobacterales* carriage in dogs were antimicrobial use (28 of 40), age (24), sex (22), hospitalization (19), and feeding raw diet (14). Of these, antimicrobial use was the most common risk factor significantly associated with AMR-*Enterobacterales* (19/28), followed by raw diet (9/14) and hospitalization (8/19). Our synthesis emphasized the importance of increasing awareness regarding the prudent use of critically important antimicrobials (CIAs), such as fluoroquinolones, in companion animal practices, strengthening infection prevention and control procedures in veterinary clinics and hospitals and educating caregivers about the potential risks of feeding raw diets in order to reduce the burden of AMR-bacteria in dogs.

## 1 Introduction

Antimicrobial resistance (AMR) has been listed among the top 10 global public health threats by the World Health Organization (WHO) (1). According to a review published in 2019, AMR directly caused more than 1.2 million deaths and was indirectly associated with nearly five million deaths in humans (2). In the absence of effective control measures, deaths due to AMR could reach 10 million people annually by 2050 (3).

Antimicrobial resistance is a critical One Health issue due to the extensive use of antimicrobials and the potential transmission of resistant bacteria and their genes between humans, animals, and their ecological niche (4, 5). Companion animals, particularly dogs and cats, have been identified as potential reservoirs and carriers of AMR-bacteria mainly due to the frequent administration of antimicrobial drugs in small animal practices and their close contact with humans (6). AMR-bacteria with identical or similar genotypes have been isolated from companion animals and their caregivers, indicating their likely transmission between humans and companion animals (7–9). This source of AMR-bacteria is of growing concern because the pet population is increasing globally, strong bonds are formed between animals and their caregivers, and they are often treated as family members with frequent close physical contact (10, 11).

The mechanisms of AMR selection and spread vary among different bacterial species and can be complex and multifactorial (12). The order *Enterobacterales*, includes *Escherichia coli* and *Klebsiella pneumoniae*, which are classified in the critical priority group by WHO due to their significant AMR threat to public health and high prevalence (1). These organisms pose a considerable challenge in the global fight against AMR, warranting focused monitoring and research to mitigate their negative impact on animals and public health.

The primary risk factors identified for the carriage or infection with AMR-*Enterobacterales* in dogs include antimicrobial use, feeding raw meat diets, and hospitalization (13–15). Some studies have also identified age, sex, and breed as potential risk factors; however, there are conflicting findings among some studies and the factors investigated vary. Identifying important risk factors contributing to AMR-*Enterobacterales* carriage is necessary to better understand their epidemiology and to develop tailored prevention and control strategies. The objectives of this systematic review were to compile published evidence on risk factors for the carriage of AMR-*Enterobacterales* in dogs reported worldwide and to identify relevant knowledge gaps for directing future research.

## 2 Methods

This systematic review was conducted according to the Preferred Reporting Items for Systematic Reviews and Meta-Analyses (PRISMA) guidelines (16).

### 2.1 Eligibility criteria

Studies that reported any risk factors for carrying or detecting AMR-*Enterobacterales* in dogs worldwide were included in our systematic review. The search was limited to peer-reviewed studies published in English. There were no date or geographical limitations for inclusion. Studies involving dogs only or dogs together with other animal species (mixed species) were included; however, the relevant data were extracted for dogs only. Studies on non-*Enterobacterales* bacteria, published in languages other than English, purely experimental, reviews, and gray literature were excluded.

### 2.2 Search strategy

The last search for eligible articles was performed in December 2023 using three electronic databases (PubMed, CABi, and Scopus) and three main domains with relevant search terms ((dog OR dogs OR canine^*^) AND (antimicrobial resistan^*^ OR AMR OR antibiotic resistan^*^) AND (Risk factor^*^ OR protective factor^*^)). The initial search string was kept wide to capture all possibly relevant articles, and an expert librarian at City University of Hong Kong was consulted to confirm and adjust the string to be compatible with each database.

### 2.3 Screening and study selection

**Figure 1:**
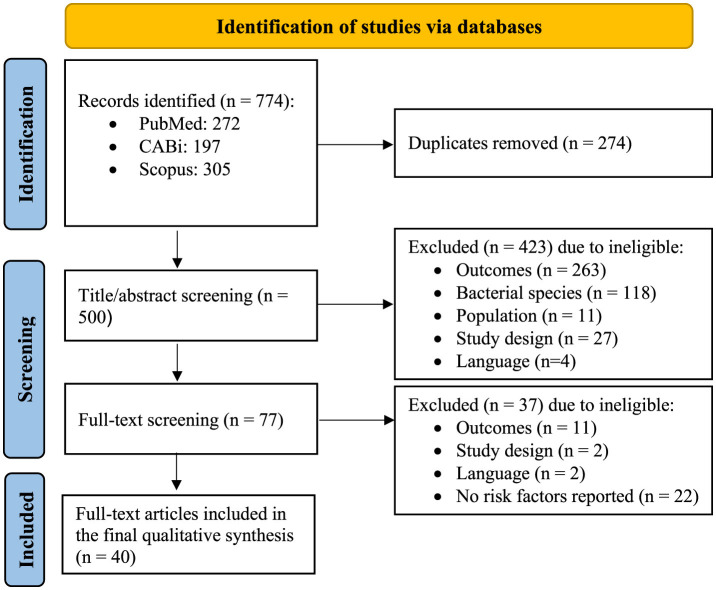
PRISMA flowchart indicating the study selection process (adapted from http://prisma-statement.org/PRISMAStatement/FlowDiagram.aspx).

In the initial search, identified studies from the three databases were exported to EndNote V 20.6 (Clavariate Analytics, 2020) and duplicates were removed. The remaining studies were uploaded to Rayyan QCRI software (http://rayyan.qcri.org) (17), and each study was screened by title/abstract, followed by a full-text review of each identified eligible study to ensure relevance. Two reviewers independently conducted the screening and selection process. Any disagreements were resolved by a third independent reviewer. All steps of the study screening and selection process are summarized in Figure 1.

### 2.4 Data extraction

The following variables and information were extracted from the body and Supplementary material of eligible studies and entered into a data extraction table in Microsoft Excel™ designed for this study: (1) General study characteristics (title, authors, journal name, year of publication, country study was performed, objectives), (2) Methodology (study type, study duration, sampling methods), (3) Animal population characteristics (species, number, sex, age, breed, neutering status), (4) Bacterial species isolated, (5) AMR patterns (e.g., ESBL), (6) Antimicrobial susceptibility test (AST) conducted, (7) AMR genes detected, (8) Statistical analysis conducted and (9) Factors investigated (risk and protective factors investigated).

### 2.5 Classification of risk factors

All investigated predictor variables for AMR-*Enterobacterales*, including risk/protective factors (hereafter, referred to as “risk factors”), extracted from the final selected studies were classified into nine main categories based on their similarities in order to provide clear summary assessments (Table 1). Most studies investigated a large number of predictor variables and were included in multiple risk factor categories. The main categories, subcategories, and statistical significance of associations between reported risk factors and the occurrence of AMR-*Enterobacterales* are summarized in Table 1, and with more details (e.g., all studied predictor variables per subcategory) in Supplementary Table 1.

**Table 1:** Defined classifications for reported variables in the 40 selected studies with the number of studies investigating the factors and those finding statistically significant associations with the carriage of AMR-*Enterobacterales* in dogs.

**Category**	**Subcategory**	**No. of studies investigated the factors**	**No. of studies finding significant association/s**	**References for significant risk factors**
1. Dog information	Age group	24	2	(40, 63)
	Sex	22	2	(31, 64)
	Breed classification	11	2	(62, 63)
	Body weight/condition	6	1	(35)
	Neuter status	4	1	(32)
	Purpose	4	-	
	Source	6	1	(31)
2. Caregiver information	Dog caregiver's general information	10	1	(35)
	Health-related information	9	1	(35)
3. Household information	Household size and type	4	-	(50)
	Multi-animal/species household	11		(35)
	Husbandry	17	1	(24)
4. Outdoor factors	Walking	11	1	(75)
	Proximity to animal/food facilities	1	1	(14)
	Contact with other animals	6	2	(24, 28)
	Feces management	2	-	
	Access to trash	2	1	(19)
	Water access	6	2	(41, 76)
5. Diet	Raw diet	14	9	(13, 20, 24, 27, 40–44)
	Commercial diet	12	2	(24, 44)
	Treats	6	1	(13)
	Homemade food	5	1	(62)
	Time on the current diet	1	-	
6. Medical history	Hospitalization information	19	8	(15, 26, 34, 36–39)
	Medical procedures and interventions	11	3	(38, 44, 50)
	Medical conditions	15	3	(23, 35, 77)
7. Antimicrobial use	Antimicrobial use	28	19	(14, 15, 22–28, 30–32, 34, 36, 39, 41, 43)
8. Other medications	Any type of medication other than antimicrobials	8	2	(25, 36)
9. Other factors	Any other type of exposure not mentioned in the other categories	12	4	(33, 35, 62, 74)

### 2.6 Quality assessment of included studies

The quality of the included studies was evaluated using the guide notes in the Cochrane Handbook for Systematic Reviews of Interventions (18), which were adapted for our study types. This is a domain-based evaluation including 10 main quality assessment domains (aim, study population, study power, masking, case definition, diagnostic classification, indication of precision, completeness of results, believable conclusions, and consistency). Critical appraisals were made separately for each domain. Risk-of-bias for each domain was defined as high, low, or unclear, based on a detailed review of each selected study.

## 3 Results

### 3.1 Study selection

In total, 774 articles were identified in our initial search of the three databases, including 272 in PubMed, 197 in CABi, and 305 in Scopus. The workflow and corresponding numbers of the articles at each stage are summarized in Figure 1. After removing duplicates, 500 unique articles remained for title/abstract screening. Subsequently, 77 articles were selected for full-text review, of which, 37 were excluded due to: (1) not reporting any risk factors (22/37), (2) irrelevant outcomes (11/37), (3) study design (2/37), and (4) language (2/37) (Figure 1). Finally, 40 studies were eligible for complete data extraction.

### 3.2 Characteristics of included studies

All 40 studies included in the final qualitative synthesis were observational, including 29 cross-sectional, six cohort, and five case-control studies. All studies were published between 2006 and 2023 and were most frequently conducted in Europe (16/40), followed by North America (8/40), Asia (8/40), South America (4/40), Australia (3/40), and Africa (1/40).

Most studies (26/40) followed a non-random sampling strategy, and 10 reported random sampling for participant selection. Four studies did not provide explicit details about sampling methods (19–21). Age of the studied dogs ranged between 1 month and 17 years.

*E.coli* was the most frequently investigated and isolated bacteria in these studies (30/40), followed by *Klebsiella* (5/40), *Salmonella* (3/40), *Citrobacter* (3/40). Nine studies (of 40) reported the concurrent isolation of multiple bacterial species as follows: 3 reported concurrent *E. coli* and *Salmonella*; 3 others isolated *E. coli* and *Klebsiella*; one study detected *E. coli* and *Citrobacter*; one study *E. coli, Klebsiella, Citrobacter* and *Morganella;* and the remaining one reported *E. coli, Klebsiella, Citrobacter, Proteus*, and *Enterobacter*. No studies reported the detection of *Shigella, Yersinia*, or *Serratia*.

The most commonly reported AMR type was extended-spectrum beta-lactamase (ESBL)-producing *E. coli* (19/40). Eight studies reported multidrug resistant (MDR)-*E. coli*. All studies conducted AST, including disk diffusion (22/40) and minimum inhibitory concentration (MIC) (18/40). Over half (24/40) used breakpoints recommended by the Clinical and Laboratory Standards Institute guidelines (CLSI), while 4/40 used the European Committee on Antimicrobial Susceptibility Testing guidelines (EUCAST), 7/40 reported both EUCAST and CLSI, 3/40 applied the British Society for Antimicrobial Chemotherapy guidelines (BSAC), and 2/40 did not report using any specific guidelines.

### 3.3 Reported risk factors

The most commonly investigated predictor variables for AMR-*Enterobacterales* carriage in dogs were antimicrobial use (28/40), age (24/40), sex (22/40), hospitalization (19/40) and raw diet (14/40) (Table 1). The most frequent statistically significant risk factor identified was previous antimicrobial use (19/28). Five studies reported previous use of antibiotics (without specifying the antibiotic classes) to be significantly linked with ESBL- and carbapenem-resistant-*E. coli* (22–26). With respect to the time frame of antibiotic use, six studies revealed a positive association between the timing of antimicrobial administration (from 10 days to one year) with ESBL, ampicillin-resistant (AmpC), and 3GCR-*E. coli* carriage (14, 27–31).

For specific antimicrobial classes, seven studies investigated specific classes of antimicrobials, of which, four reported the prior use of fluoroquinolones as significantly associated with ESBL, quinolone-resistant, and MDR-*E. coli* (15, 32–34). One study associated fluoroquinolones, cephalosporins, penicillin beta-lactamase with 3-GCR, AmpC-producing, and fluoroquinolone-resistant *E. coli* (35). One study reported fluoroquinolones and cephalosporins to be associated with ESBL-*E. coli* (32), while another study associated using cephalosporins and nitroimidazole with MDR-*E.coli* (36).

Lastly, one study showed that antimicrobial use in any household animal species in the past 6 months and purchase of antimicrobials from a pet food store were significantly associated with 3-GCR and ESBL-*E. coli* (14).

Other common risk factors found to be significantly associated with AMR- *Enterobacterales* carriage in dogs were raw diet (9/14) and hospitalization (8/19). Long duration of hospitalization (≥3 days) increased the risk of acquiring ESBL-*E. coli*, ESBL-*K. pneumoniae* and MDR-*E. coli* (4/8) (15, 36–38). In addition, being hospitalized and attending a referral consultation were found to be risk factors for ESBL- and AmpC-producing *E. coli*, respectively (3/8) (34, 35, 39). Feeding raw meat to dogs was associated with 3-GCR- and ESBL-*E. coli* in nine studies (13, 20, 27, 40–44), of which, two reported a significant association between raw chicken consumption and AMR *Salmonella* and *E.coli* (43, 44). However, no significant association was identified between the frequency of raw food consumption and the occurrence of AMR-bacteria carriage in these studies.

The odds ratios and corresponding confidence intervals for each factor (statistically significant or not), where reported in the 40 studies, are included in Supplementary Table 2.

### 3.4 Quality assessment (risk-of-bias)

**Figure 2:**
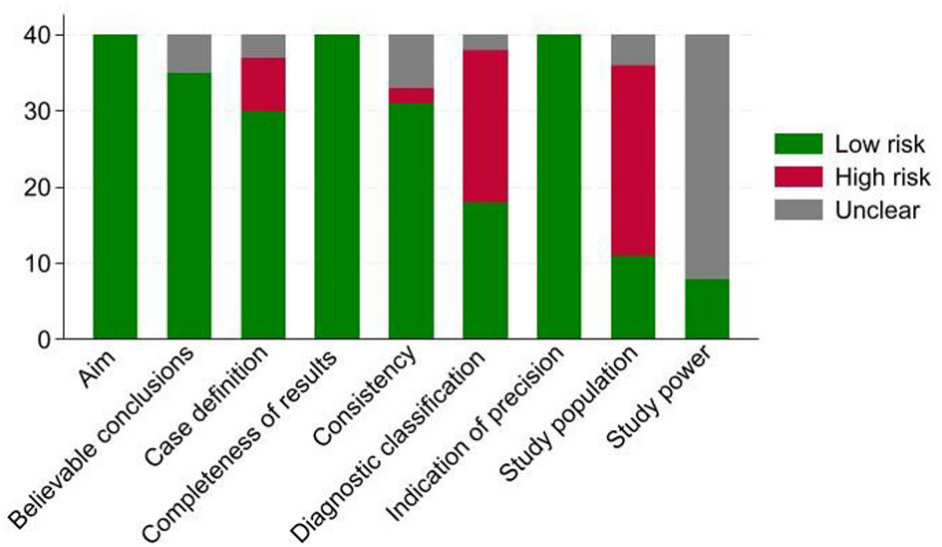
Frequency distribution of our 40 systematically reviewed studies categorized as having high, low, or unclear risk-of-bias by each domain; the framework was adapted from (78).

The domain “masking” was removed from our analysis because it is not relevant to observational studies. The number of articles with low, unclear, or high risk-of-bias (of 40) for each domain is summarized in Figure 2. The main factors leading to a high risk of bias included non-random, small, convenient sampling (62.5% of studies), and the lack of information regarding the potential variables that could lead to bias (50%). In addition, in 80% of the studies, authors did not report how the sample size was calculated; therefore, the risk of bias regarding the study power was unclear.

## 4 Discussion

To the best of our knowledge, this is the first comprehensive review synthesizing the risk factors associated with the carriage of AMR-*Enterobacterales* in dogs on a global scale. By compiling a diverse range of risk factors, we aimed to provide an overview of risk factors that could contribute to the acquisition and spread of AMR in dogs worldwide. The most commonly reported risk factor was antimicrobial use. Fluoroquinolones, cephalosporins, penicillin beta-lactamases, and nitroimidazoles were among the antimicrobial classes significantly associated with the third-generation cephalosporin-resistant, AmpC-producing, and quinolone-resistant-*E. coli* (35). This finding is likely due to the extensive use of these drugs in companion animal practices and veterinary medicine in general (45). In fact, fluoroquinolones are regarded as “highest priority, critically important antimicrobials” (CIAs) by the WHO, World Organization for Animal Health, and European Medicines Agency with high likelihood and consequences (risk) of AMR development (46, 47).

In addition to antimicrobial use, our study revealed several other medically important risk factors that may impact the carriage of AMR-*Enterobacterales* in dogs. Hospitalization was the third most common risk factor reported. Prolonged hospital stay is associated with an increased probability of exposure to contaminated surfaces and objects, contact with other hospitalized animals and interaction with the hospital personnel (48). These factors are known to contribute to the transmission of AMR-bacteria in human-based studies as well (49). Two studies have conducted an in-depth analysis of risk factors in veterinary hospitals, examining specific hospital units, such as oncology, neurology, orthopedics, cardiology and others (37, 50). Among these factors, exposure to anesthesia, surgery, diagnostic imaging and intubation were found to have a significant association with the acquisition of carbapenem-resistant and *bla*_NDM − 5_ carbapenem-resistant *E. coli* (50). These findings are consistent with other published studies in humans, which have highlighted the significance of medical services and devices in patient care as a contributing factor to the transmission of infections and AMR-bacteria (51, 52). However, unlike in human healthcare settings, in veterinary medicine, reusing some devices, such as endotracheal tubes, is claimed to be relatively common which can pose a serious risk regarding the acquisition and spread of AMR-pathogens (50). These findings call for strengthening infection prevention and control procedures, and implementing active surveillance in veterinary clinics and hospitals, particularly in developing countries.

A significant association between chronic or recurrent diseases and MDR-*E. coli* and ESBL-*E. coli* was reported in two studies (out of 14), where dogs with chronic urinary tract infections (UTIs) previously treated with antimicrobials were found to be eight times more likely to carry MDR-*E. coli* compared to dogs with acute UTIs and no history of prior antimicrobial treatment (23, 35). These findings suggested that the prolonged use of antimicrobials for the treatment of chronic diseases can substantially contribute to an increased risk of acquiring AMR-*E. coli* infections. Interestingly, annual vaccination and heartworm preventive treatment within the past 6 months demonstrated a significant protective effect against AMR-*Salmonella* spp. and AMR-*E.coli* (53). Other studies have not reported this association, suggesting that these variables may have served as proxies for consistent exposure to veterinary care (44).

Some studies have shown that lifestyle and dietary choices can significantly influence resistance patterns. For example, nine (out of 14) studies that investigated raw diet, found a significant association between the consumption of raw meat and AMR-*Salmonella* spp., clavulanate-amoxicillin and third-generation cephalosporin-resistant *E.coli*. Consuming raw poultry meat was reported in two of these studies (43, 44). The high prevalence of ESBL-*E.coli* in chicken meat has been well-documented (54, 55). The administration of antimicrobials in food-producing animals, for growth promotion and/or disease treatment, has been identified as a significant risk factor in the spread of AMR-bacteria within the food chain (56, 57). In addition, the presence of environmentally persistent *Salmonella* in the pet food factory setting has been well documented, and biofilm formation may serve as a potential mechanism contributing to their long-term survival (58). The beliefs held by some pet owners that raw food is more “natural” and “healthier” compared to processed commercial foods may pose a risk to the animals through the acquisition and spread of various pathogens and AMR-bacteria (59, 60). This raises public health concerns due to the often frequent close contact between owners, pets, and their shared environment. Collective efforts to educate caregivers about the potential risks of raw feeding have been made in some developed countries by their respective veterinary associations; however, not adequate at a global scale (61). Caregivers should be educated on the potential risks of giving raw meat or meat products to their pets. In contrast, feeding supplements (e.g., vitamins and minerals), herbal products, proprietary dog treats and homemade cooked diets showed a protective effect against ciprofloxacin-resistant and MDR-*E.coli* (13, 24, 44, 62). The authors highlighted the scarcity of research investigating the protective effects of food supplements, particularly in small animal medicine. However, it is assumed that caregivers, who provide supplements to their dogs, care more about their health (i.e., a confounding factor effect). The observed protective associations of dog treats and homemade cooked diets can be attributed to the elimination of *E. coli* in the production process, particularly when heat treatment is used (24).

After antimicrobial use, age was the most commonly investigated factor (24/40). A higher prevalence of AMR was found in *E. coli* isolated from dogs older than 10 years compared to those isolated from younger dogs (1–3 years old) (27, 63). This was attributed to suppressed immunity in geriatric dogs and selection pressure from prior antimicrobial use in younger dogs (63). Sex was also a commonly investigated factor (22/40), with only two studies reporting significant associations with AMR-bacteria (53, 64). One study demonstrated that female dogs were at higher risk of carrying pAmpC-producing *E. coli* (31), potentially due to a higher incidence of bacterial infections in the lower urinary tract. In contrast, the other study reported a significantly higher prevalence of cephalosporin-resistant *Enterobacterales* in males (64), with no explanation for this observation. The fact that most studies (20/22) did not find any significant associations indicates that age and sex are not likely to be primary determinants for acquiring and spreading AMR-bacteria in dogs. However, in humans, a recent study conducted across 29 European countries found that the occurrence of methicillin-resistant *Staphylococcus aureus* increased with age, while the occurrence of aminopenicillin resistance in *E.coli* decreased with age (65). The authors also concluded that men generally exhibited a higher risk of antimicrobial resistance compared to women. These findings may suggest that future interventions aimed at reducing the burden of AMR in animals and humans should take into account the significant variations in AMR patterns and prevalence observed across different age groups and between sexes.

Two studies (out of 11) reported breed as a risk factor, indicating a higher prevalence of AMR-*E.coli* among terriers, herding dogs, and large mixed-breed dogs compared to other breeds (62, 63). The reported high prevalence was attributed to certain lifestyle aspects among large mixed–breed dogs, although no specific aspects were explicitly mentioned. In addition, 20% of these dogs were obtained from a humane society, where animals are in close contact, leading to a higher likelihood of acquiring AMR-bacteria (63). In another study, smaller dogs were also less likely to have resistant isolates of AmpC-producing *E. coli* compared to heavier ones (13). Dogs from shelters or breeders had also a higher risk of carrying AmpC-producing *E. coli*, potentially due to frequent antimicrobial use in breeding kennels and common amoxicillin-clavulanate acid use in veterinary medicine (31). Neutering status (i.e., being intact) was significantly associated with the carriage of the mobile colistin resistance gene, *mcr-1* (32). They explained hormonal and behavioral changes resulting from ovariectomy/castration were expected to affect the composition and diversity of gut microbiota, potentially impacting the colonization of AMR genes; the opposite of what was observed in this study. However, intact dogs are expected to be more active and more likely to contact other dogs than neutered dogs in estrus, which could have encouraged them to acquire mcr-1-positive bacteria from other dogs and the environment (32).

Environmental factors, such as walking in the countryside, contact with puppies, access to compost, commercial poultry odors detected, living within 5 km of the nearest commercial food animal facilities, direct contact with livestock playing in the river, and having access to ditches/puddles were found to have a positive association with the carriage of AMR-*Enterobacterales* from the study dogs in seven of the reviewed studies. Environmental contamination through livestock farming practices, such as the use of poultry feces as fertilizers or the contamination of shared water sources, can contribute to the spread of AMR-bacteria among domestic animals (66). Playing in rivers and having access to puddles/ditches was positively associated with the third-generation cephalosporin (3GC)-resistant *E.coli* and ESBL-*E.coli*. Previous research has shown that contaminated water sources serve as distinct environments for the spread of AMR genes among pathogens (67). Access to compost was linked with multiclass-resistant *E.coli* in one study (62). However, it was not specified whether dogs consumed the compost or only had contact with it and the level of exposure was also unknown. Compost may contain animal by-products and the feces of wild animals, which could harbor AMR-bacteria (62).

A positive correlation for the carriage of the colistin resistance gene, *mcr-1*, was shown between dogs and their caregivers in one study (32), in which, dogs consuming human food (shared with their owners) had a higher prevalence of *mcr-1* (24.5%) compared to those eating commercial dog food (14.3%). This finding highlighted the potential role of shared dietary habits and living areas in the transmission of the *mcr-1* gene (32). Moreover, previous research has also shown the presence of genetically similar methicillin-resistant *Staphylococcus pseudintermedius* in patients, contact animals, and environmental samples within the same household (68). However, further studies should focus on the sources and genetic similarities of AMR bacteria between pets, owners, and their environment for a better understanding of the direction of transmission.

Administration of corticosteroids 1 month prior to the hospitalization was found to be significantly associated with ESBL-*E.coli* carriage in dogs (25). In humans, a significant association between corticosteroid use and carriage of ESBL-*K. pneumoniae* was reported in patients with rheumatic autoimmune diseases (69); however, the authors did not explain the possible reasons behind this association. Additional studies are required to provide further insights into the association between using corticosteroids and AMR. Moreover, the interval time between admission to the hospital and treatment with NSAIDs demonstrated a protective effect against the development of MDR-*E.coli* in one study (out of two) (36). While the authors did not provide any reasons for this effect, previous research in humans suggests that certain NSAIDs (acetylsalicylic acid, diclofenac, ibuprofen) have anti-biofilm activities in concentrations found in human pharmacokinetic studies (70–72). However, most of these studies were conducted *in vitro* and tested non-clinical bacterial isolates, which may not represent the bacterial populations that cause clinically relevant infections. Despite the promising potential of NSAIDs for controlling biofilm-related infections, there is a lack of research, particularly controlled clinical trials to support their use as anti-biofilm agents (73).

Attending dog daycare was associated with the carriage of AmpC producing *E. coli* in one study (62). The authors explained that while previous research had shown dogs within the same breeding kennel shed bacteria with greater similarity in resistance patterns, no studies had specifically investigated the AMR spread in dog daycare centers.

Coprophagic habits were also positively associated with ESBL-*E. coli* gut colonization and MDR-*E. coli* in two studies (33, 74). This common behavior in dogs allows the ingestion of gut microflora, including AMR-bacteria. Feces from animals undergoing antimicrobial treatments may contain drug residues that may further contribute to the selection for and spread of AMR-bacteria. This highlights the important role of veterinarians in hospitals and clinics in educating pet caregivers to prevent and control this behavior.

While this study was designed to transparently and objectively assess the selected studies using the risk-of-bias domains, there were some potential limitations, including small sample sizes, which were frequently reported as a study-level limitation. Many studies either did not conduct or report a sample size calculation. This was a common factor limiting the statistical power of the studies in determining significant risk factors. Another common limitation was the lack of clear information about sampling strategies. Based on the available information, we assumed that a convenient sampling method was applied in these studies, which may substantially limit the internal and external validity (generalizability) of some findings. Some studies also lacked detailed clinical histories, including important factors such as prior antimicrobial use, which could have confounded their results. Our final selection included studies conducted in all continents. However, inevitably, studies from English-speaking countries are relatively overrepresented because of our logistical limitations to include non-English literature. This may somewhat decrease the generalizability of our compiled findings to geographical locations with particular management conditions. The risk factors investigated and their statistical significance could have been affected in the individual studies by including dogs from a wide range of health states. In the diseased dog populations, compared to healthy ones, it is presumed that the likelihood of acquiring and disseminating AMR-bacteria can be higher due to various factors, such as the higher use of antimicrobials and exposure to hospital environments. Lastly, we need to emphasize that our approach of relying on the number of reports (i.e., agreement in findings of different studies) and the significance of reported factors can to some extent simplify the importance of these risk factors. Although we attempted to account for the magnitude of reported odds ratios, along with their corresponding confidence intervals (see Supplementary Table 2), clear inconsistency in reporting and substantial heterogeneity in the study designs and statistical methods precluded conducting a robust meta-analysis on the extracted odds ratios for the commonly investigated risk factors. The readers must keep this limitation in mind when interpreting our findings.

## 5 Conclusions

Based on our systematic review, a fair amount of evidence has already been published on potential risk factors associated with the carriage of AMR-*Enterobacterales* in dogs. Our summary has implications for small animal veterinary practitioners, public health authorities, and researchers who are actively involved in addressing the challenge of AMR. Antimicrobial use, feeding raw diets, and hospitalization were the most significant factors reported consistently in the reviewed literature. This highlights the need for implementation and maintaining high standards of antimicrobial stewardship to reduce our reliance on priority classes of antimicrobials and educate pet caregivers on proper diet for their dogs. While several other risk factors have been identified such as age, sex, breed, dog owner's related factors, or the use of medications other than antimicrobials, their potential associations with the carriage of AMR-*Enterobacterales* are somewhat conflicting or poorly understood. This was either due to the low number of studies specifically designed to address these factors or shortfalls in the sample size and/or study design prohibiting robust statistical analyses. Further studies directed at such potential risk factors are warranted as a comprehensive understanding of the underlying risk factors is crucial for the development of targeted, effective preventive strategies and interventions. Lastly, our quality assessment of the reviewed literature calls for maintaining high standards in study design and peer-review processes by the scientific journals.

## Data Availability

The original contributions presented in the study are included in the article/Supplementary material, further inquiries can be directed to the corresponding authors.
