# Supplementary material for: Risk factors for antimicrobial-resistant Enterobacterales in dogs: a systematic review
Source: Front Vet Sci. 2024 Oct 7;11:1447707. doi: 10.3389/fvets.2024.1447707 (PMC11493919; doi:10.3389/fvets.2024.1447707)
Supplement: Supplementary file 1 [file Table_1.docx]

**Table S1.** Defined classifications for reported predictor variables in the 40 selected studies with the number of studies investigating the factors and those finding statistically significant associations with the carriage of AMR-*Enterobacterales* in dogs.

| **Category** | **Subcategory** | **Predictor variables investigated** | **No. of studies investigated the factors** | **No. of studies finding significant association/s** | **References for significant risk factors** |
| --- | --- | --- | --- | --- | --- |
| **1. Dog information** | Age group | - Age | 24 | 2 | [40, 63] |
|  | Sex | - Sex | 22 | 2 | [31, 64] |
|  | Breed classification | - Breed | 11 | 2 | [62, 63] |
|  | Body weight/condition | - Weight | 6 | 1 | [35] |
|  | Neuter status | - Neuter status | 4 | 1 | [32] |
|  | Purpose | - Working | 4 |  |  |
|  |  | - Breeding |  |  |  |
|  |  | - Hunting |  |  |  |
|  | Source | - Pet store | 6 | 1 | [31] |
|  |  | - Breeder |  |  |  |
|  |  | - Shelter |  |  |  |
|  |  | - Farm |  |  |  |
|  |  | - Humanitarian society |  |  |  |
|  |  | - Imported |  |  |  |
| **2. Caregiver information** | Dog caregiver’s general information | - Age | 10 | 1 | [35] |
|  |  | - Gender |  |  |  |
|  |  | - Level of education |  |  |  |
|  |  | - Living location |  |  |  |
|  |  | - Occupation |  |  |  |
|  | Health-related information | - Previous antimicrobial use | 9 | 1 |  |
|  |  | - History of diarrhea/vomiting |  |  |  |
|  |  | - Contact with diarrhea patients |  |  |  |
|  |  | - Previous hospitalization |  |  |  |
|  |  | - Vaccination |  |  |  |
|  |  | - Presence of immunocompromised persons in the household |  |  |  |
|  |  | - Healthcare visit |  |  |  |
|  |  | - Knowledge about antimicrobial use |  |  |  |
| **3. Household information** | Household size and type | - Number of members | 4 |  | [50] |
|  |  | - Type (urban, suburban, small town rural, non-farm rural) |  |  |  |
|  | Multi-animal/species household | Multi-animal at home | 11 |  | [35] |
|  | Husbandry | - Habitat type (indoor/outdoor/mixed) | 17 | 1 | [24] |
|  |  | - Dog’s access to bed/furniture |  |  |  |
|  |  | - Playing in bed |  |  |  |
|  |  | - Licking owner’s face |  |  |  |
|  |  | - Access to cat litter |  |  |  |
|  |  | - Caged or confined |  |  |  |
| **4. Outdoor factors** | Walking | Walking on: | 11 | 1 | [75] |
|  |  | - Streets |  |  |  |
|  |  | - Parks |  |  |  |
|  |  | - Beaches |  |  |  |
|  |  | - Countryside |  |  |  |
|  |  | - Farmlands |  |  |  |
|  |  | - Supervised/unsupervised |  |  |  |
|  |  | - In contact with other animals during walk |  |  |  |
|  | Proximity to animal/food facilities | - <1km | 1 | 1 | [14] |
|  |  | - 1-2km |  |  |  |
|  |  | - >2-5km |  |  |  |
|  |  | - 5–10km |  |  |  |
|  |  | - 10–20km |  |  |  |
|  |  | - >20km |  |  |  |
|  | Contact with other animals | - Contact | 6 | 2 | [24, 28] |
|  | Faeces management | Leave in: | 2 | - |  |
|  |  | - the trash, |  |  |  |
|  |  | - place in the yard, |  |  |  |
|  |  | - store |  |  |  |
|  | Access to trash | To trash/dead animals/compost | 2 | 1 | [19] |
|  | Water access | To any water source outside home | 6 | 2 | [76, 77] |
| **5. Diet** | Raw diet | Any type of raw food and the frequency of eating raw food | 14 | 9 | [13, 20, 24, 27, 40, 42-44, 76] |
|  | Commercial diet | - Dry/wet, cooked | 12 | 2 | [24, 44] |
|  |  | - Dry conventional |  |  |  |
|  |  | - Supplements |  |  |  |
|  |  | - Source |  |  |  |
|  |  | - Probiotics |  |  |  |
|  |  | - Herbal products |  |  |  |
|  | Treats | Any types of treats | 6 | 1 | [13] |
|  | Homemade food | - Homemade cooked | 5 | 1 | [62] |
|  |  | - Human food use |  |  |  |
|  |  | - Cooked table scraps |  |  |  |
|  | Time on the current diet | -Time duration | 1 | - |  |
| **6. Medical history** | Hospitalization information | - Admission to hospital & duration of stay | 19 | 8 | [15, 26, 34, 36-39] |
|  |  | - Number of times hospitalized |  |  |  |
|  |  | - Clinic/hospital/referral consultation |  |  |  |
|  |  | - Time at risk while hospitalized |  |  |  |
|  |  | - The interval between admission and treatment |  |  |  |
|  | Medical procedures and interventions | Exposure to: | 11 | 3 | [38, 44, 50] |
|  |  | - Surgery |  |  |  |
|  |  | - Neurology unit |  |  |  |
|  |  | - Nursing care unit |  |  |  |
|  |  | - Intensive care unit |  |  |  |
|  |  | - Anaesthesia |  |  |  |
|  |  | - Urine and blood collection |  |  |  |
|  |  | - Physical therapy |  |  |  |
|  |  | - Diagnostic imaging |  |  |  |
|  |  | - Emergency |  |  |  |
|  |  | - Soft tissue and internal medicine |  |  |  |
|  |  | - Orthopaedics |  |  |  |
|  |  | - Dermatology |  |  |  |
|  |  | - Oncology |  |  |  |
|  |  | - Endotracheal intubation |  |  |  |
|  |  | - Catheterization |  |  |  |
|  |  | - Computed tomography scan |  |  |  |
|  |  | - Vaccination, deworming |  |  |  |
|  | Medical conditions | - Acute/chronic diseases | 15 | 3 | [23, 35, 78] |
|  |  | - Illness severity |  |  |  |
|  |  | - Diarrhea |  |  |  |
|  |  | - Vomiting |  |  |  |
|  |  | - Urinary tract infections |  |  |  |
|  |  | - Neurological |  |  |  |
|  |  | - Cardiovascular |  |  |  |
|  |  | - Haematological |  |  |  |
|  |  | - Gastrointestinal |  |  |  |
|  |  | - Respiratory |  |  |  |
|  |  | - Hepatic |  |  |  |
|  |  | - Orthopaedic |  |  |  |
|  |  | - Ophthalmologic diseases |  |  |  |
|  |  | - Tumours |  |  |  |
|  |  | - Intoxications |  |  |  |
| **7. Antimicrobial use** | Antimicrobial use | Any antimicrobial classes, the duration and source of purchase (veterinarian, pet food store) | 28 | 19 | [14, 15, 22-28, 30-32, 34, 36, 39, 43, 76] |
| **8. Other medications** | Any type of medication other than antimicrobials | - Non-steroidal anti-inflammatory drugs (NSAIDs) | 8 | 2 | [25, 36] |
|  |  | - Steroids, |  |  |  |
|  |  | - Opioids |  |  |  |
|  |  | - Chemotherapy |  |  |  |
| **9. Other factors** | Any other type of exposure not mentioned in the other categories | - Kennel or hotel visit/access, | 12 | 4 | [33, 35, 62, 74] |
|  |  | - Grooming services |  |  |  |
|  |  | - Abroad travels |  |  |  |
|  |  | - Time the dog was owned, |  |  |  |
|  |  | - Hygiene status (e.g., not clean enough, somewhat clean, clean) |  |  |  |
|  |  | - Coprophagia |  |  |  |
|  |  | - Household wealth |  |  |  |
|  |  | - Presence of a toilet in the household |  |  |  |
|  |  | - Presence of owner at the point of admittance to the shelter |  |  |  |
|  |  | - Distance from household to the nearest urban centre |  |  |  |
|  |  | - Sampling source and season |  |  |  |
